# Prevalence of overweight and obesity in 15.8 million men aged 15–49 years in rural China from 2010 to 2014

**DOI:** 10.1038/s41598-017-04135-4

**Published:** 2017-07-10

**Authors:** Yuan He, An Pan, Yuanyuan Wang, Ying Yang, Jihong Xu, Ya Zhang, Dujia Liu, Qiaomei Wang, Haiping Shen, Yiping Zhang, Donghai Yan, Zuoqi Peng, Frank B. Hu, Xu Ma

**Affiliations:** 1 National Research Institute for Health and Family Planning, Beijing, China; 2 Research Center for Population Health and Risk Assessment, National Human Genetic Resources Center, Beijing, People’s Republic of China; 3 Department of Epidemiology and Biostatistics, Ministry of Education and State Key Laboratory of Environmental Health (Incubating), School of Public Health, Tongji Medical College, Huazhong University of Science and Technology, Wuhan, Hubei China; 4 Department of Maternal and Child Health, National Health and Family Planning Commission, Beijing, People’s Republic of China; 5 Department of Nutrition and Department of Epidemiology, Harvard T.H. Chan School of Public Health, Boston, MA USA

## Abstract

Obesity has been increasing worldwide. Data on obesity status among men of reproductive age are scarce. This study aimed to assess the national prevalence and trend of overweight and obesity among reproductive-age men in rural China. In the nationwide population-based study, data were obtained from the National Free Preconception Health Examination Project in rural China from 2010 to 2014. Weight and height were measured in 16 161 982 men aged 15–49 years and their female partners (15 997 739 participants aged 15–49 years) before conception, and body mass index (BMI) was calculated. We found that the prevalence of combined overweight and obesity among men was 33.8% (33.7–33.9%) according to Chinese criteria (BMI ≥ 24.0 kg/m^2^), the corresponding rates of obesity were 6.3% (6.2–6.4%; BMI ≥ 28.0 kg/m^2^), respectively. The prevalence varied in different ethnic groups, geographic regions, age, education and GDP levels, and increasing trend was observed over the 5-year study period. In addition, 45.8% of couples had at least one of them as overweight or obesity. About one third of men aged 15–49 years before conception in rural China are overweight or obese. Overweight/obesity clusters in families, which highlights the importance of family involvement of early prevention of obesity in China.

## Introduction

Obesity prevalence has increased dramatically over the past several decades, and the global estimate suggests that more than one-third of adults are overweight or obese^1^. Overweight and obesity are well-established risk factors for diabetes, cardiovascular disease (CVD), certain cancers and premature death, as well as adverse reproductive consequences^2–5^. Studies have documented that maternal obesity is associated with an increased risk of infertility, pregnancy complications, and chronic diseases in the offspring^6^. However, whether paternal obesity has an effect on the pregnancy outcomes and long-term health of their children remains to be explored. Several studies have shown that male obesity is related to higher risk of infertility, and accumulating evidence also indicates the importance of paternal obesity in programming offspring obesity/metabolic phenotype through epigenetic modifications at the very early stage, even before conception^7–9^.

Substantial variability exists across the globe in the prevalence of overweight and obesity in men of reproductive age^1^. In the US, about two thirds of men aged 20–39 years were overweight or obese in 2012, although the prevalence remained stable in recent years^10^. A previous small-scale study reported that the prevalence of overweight and obesity in China tripled from 8.2% to 26.5% among men aged 18–39 years between 1993 and 2009^11^. For better assessing the paternal risks and improving pregnancy outcomes, it is critical to examine weight status of reproductive-age men before conception; however, relevant data are scarce. The purpose of this study is to provide the most recent national estimates of overweight and obesity among reproductive-age men before conception in rural China and to analyze the trends between 2010 and 2014. Data are based on measured values of weight and height from the National Free Preconception Health Examination Project (NFPHEP) database, a mega cohort of more than 15 million couples.

## Results

The detailed demographic characteristic of reproductive-age men across study years are shown in Table 1. Based on data for 16 161 982 men aged 15–49 years in rural China, the numbers of participants were almost equally distributed in the three age groups (15–29, 30–39, and 40–49 years), and majority of them (61.9%) had an education level of junior high school, while only 13.4% had college or higher degrees. Among their female partner (See Supplementary Table S1), the majority of women (77.8%) were younger than 30 years old, and other characteristics of women were similar to that of men.

**Table 1:** Sample sizes and weighted proportion of demographic characteristics of reproductive-age men in rural China between 2010 and 2014^a^.

	2010	2011	2012	2013	2014	Total
**Total**	308 383	928 606	3 656 639	5 848 388	5 419 966	16 161 982
**Age groups**						
15–29	37.9	40.1	33.4	34.7	31.7	33.9
30–39	34.0	32.2	31.1	31.8	32.7	32.0
40–49	28.1	27.7	35.5	33.5	35.6	34.1
**Ethnic origin**						
Han	96.6	91.6	89.7	90.8	91.1	90.8
Uyghur	—	3.5	2.6	2.4	2.0	2.3
Zhuang	1.7	1.2	1.9	1.8	1.8	1.8
Miao	0.2	0.8	1.2	0.9	1.1	1.0
Manchu	0.1	1.0	0.6	0.7	0.6	0.6
Mongol	—	0.6	0.7	0.6	0.5	.06
Yi	1.1	0.7	0.4	0.5	0.6	0.5
Hui	0.2	0.3	1.3	1.2	1.2	1.1
Tujia	—	0.3	1.2	0.8	0.8	0.8
Tibetan	0.2	0.1	0.4	0.5	0.4	0.4
**Region**						
North	12.1	22.1	10.3	11.5	11.9	12.1
Northeast	4.1	7.3	11.0	12.6	13.7	12.0
East	19.7	25.5	20.1	23.7	24.3	23.1
Central	13.0	13.0	18.7	16.7	15.5	16.4
South	17.0	7.0	9.5	11.7	10.0	10.4
Northwest	20.6	13.9	10.4	9.2	8.5	9.9
Southwest	19.7	11.2	19.9	14.6	16.1	16.2
**Education**						
Primary school or below	7.7	7.3	10.3	8.9	8.2	8.9
Junior high school	64.6	64.5	63.4	61.8	60.0	61.9
High school	18.7	17.2	15.8	15.4	15.7	15.8
College or higher	9.0	10.9	10.4	14.0	16.1	13.4
**GDP level**						
Tertitle 1	46.6	48.9	45.0	40.2	42.2	42.9
Tertitle 2	19.6	21.5	28.8	30.7	30.7	29.2
Tertitle 3	33.8	29.6	26.2	29.1	27.1	27.9

^a^The data are shown as percentage of participants in each year, and all proportion estimates were weighted using the China Population Census in 2010.

The overall prevalence (95% CI) of combined overweight and obesity was 33.6% (33.5–33.7%) among reproductive-age men, and the prevalence of obesity alone was 6.3% (6.2–6.4%) according to the Chinese criteria (Table 2). The prevalence of overweight and obesity was higher among men in the 30–39 and 40–49 years age group compared to those aged 15–29 years. Among our participants, the minority ethnic groups of Mongolian (43.7%) and Manchu (43.0%) had substantially higher rates of overweight and obesity than do Han ethnic group (34.0%). The prevalence varied dramatically across regions, and it was highest in the north region (43.5%), and lowest in the south (24.8%) or northwest (25.9%) regions. The prevalence increased monotonically with higher education and GDP levels. The prevalence (95% CI) according to the WHO criteria was 22.4% (22.3–22.5%) for combined overweight and obesity, and 2.5% (2.4–2.6%) for obesity (See Supplementary Table S2). The overall and the sociodemographic profiles were similar to that based on the Chinese criteria.

**Table 2:** Prevalence of overweight and obesity for reproductive-age men aged 15–49 years in rural China, 2010–2014^a^.

	Overweight and obesity			Obesity		
	Prevalence	Crude OR (95%CI)	Adjusted OR (95%CI)^b^	Prevalence	Crude OR (95%CI)	Adjusted OR (95%CI)^b^
**Total**	33.6 (33.5–33.7)			6.3 (6.2–6.4)		
**Age groups**						
15–29	26.9 (26.8–27.0)	1.00	1.00	5.4 (5.3–5.5)	1.00	1.00
30–39	37.2 (37.1–37.2)	1.61 (1.60–1.62)	1.63 (1.62–1.64)	7.4 (7.3–7.5)	1.42 (1.41–1.43)	1.42 (1.41–1.44)
40–49	37.0 (36.6–37.0)	1.58 (1.57–1.60)	1.69 (1.67–1.71)	6.1 (6.0–6.2)	1.14 (1.12–1.16)	1.21 (1.18–1.23)
**Ethnic origin**						
Han	34.0 (33.9–34.1)	1.00	1.00	6.4 (6.3–6.5)	1.00	1.00
Uyghur	33.0 (32.7–33.4)	0.96 (0.94–0.97)	1.52 (1.50–1.55)	5.2 (5.0–5.4)	0.80 (0.78–0.83)	1.51 (1.46–1.57)
Zhuang	21.5 (21.1–21.8)	0.53 (0.52–0.54)	0.65 (0.63–0.66)	2.8 (2.6–2.9)	0.42 (0.40–0.44)	0.60 (0.57–0.63)
Miao	25.0 (24.4–25.5)	0.65 (0.63–0.66)	0.93 (0.91–0.96)	3.1 (2.9–3.3)	0.47 (0.43–0.50)	0.77 (0.72–0.83)
Manchu	43.0 (42.1–43.8)	1.46 (1.41–1.51)	1.05 (1.01–1.09)	8.8 (8.3–9.2)	1.40 (1.33–1.49)	1.06 (0.99–1.12)
Mongol	43.7 (42.9–44.4)	1.50 (1.46–1.55)	1.11 (1.08–1.15)	9.6 (9.2–10.0)	1.55 (1.48–1.62)	1.13 (1.08–1.19)
Yi	23.0 (22.3–23.7)	0.58 (0.56–0.60)	1.04 (0.99–1.08)	3.9 (3.6–4.2)	0.59 (0.54–0.64)	1.26 (1.16–1.38)
Hui	28.6 (28.0–29.2)	0.78 (0.76–0.80)	1.15 (1.11–1.18)	5.9 (5.5–6.2)	0.91 (0.86–0.97)	1.40 (1.32–1.50)
Tujia	26.3 (25.7–26.9)	0.69 (0.67–0.71)	0.81 (0.78–0.84)	3.3 (3.0–3.5)	0.49 (0.46–0.53)	0.66 (0.61–0.71)
Tibetan	23.5 (22.5–24.6)	0.60 (0.56–0.63)	1.07 (1.01–1.14)	3.7 (3.1–4.3)	0.56 (0.47–0.66)	1.23 (1.04–1.46)
**Region**						
North	43.5 (43.2–43.8)	2.34 (2.30–2.37)	3.03 (2.98–3.08)	9.8 (9.6–10.0)	2.71 (2.64–2.78)	3.56 (3.47–3.66)
Northeast	40.5 (40.2–40.8)	2.07 (2.04–2.10)	2.38 (2.34–2.41)	7.4 (7.3–7.6)	1.99 (1.94–2.05)	2.48 (2.41–2.56)
East	38.9 (38.7–39.0)	1.93 (1.91–1.95)	2.06 (2.04–2.09)	8.3 (8.2–8.4)	2.27 (2.22–2.32)	2.34 (2.29–2.39)
Central	29.2 (29.1–29.3)	1.25 (1.24–1.27)	1.39 (1.37–1.40)	4.5 (4.4–4.6)	1.18 (1.16–1.21)	1.29 (1.26–1.31)
South	24.8 (24.6–24.9)	1.00	1.00	3.9 (3.8–4.0)	1.00	1.00
Northwest	25.9 (25.7–26.0)	1.06 (1.05–1.07)	1.54 (1.52–1.57)	3.8 (3.7–3.9)	0.98 (0.96–1.01)	1.49 (1.45–1.54)
Southwest	28.2 (28.1–28.4)	1.20 (1.18–1.21)	1.50 (1.48–1.52)	4.6 (4.5–4.7)	1.19 (1.16–1.22)	1.59 (1.55–1.63)
**Education**						
Primary school or below	28.5 (28.2–28.7)	1.00	1.00	4.8 (4.7–4.9)	1.00	1.00
Junior high school	32.2 (32.1–32.3)	1.19 (1.18–1.21)	1.10 (1.09–1.12)	5.7 (5.6–5.8)	1.19 (1.15–1.23)	0.97 (0.94–1.01)
High school	34.7 (34.5–34.9)	1.34 (1.32–1.36)	1.31 (1.29–1.33)	7.1 (7.0–7.2)	1.51 (1.45–1.56)	1.27 (1.22–1.31)
College or higher	41.9 (41.6–42.1)	1.81 (1.78–1.84)	1.67 (1.64–1.70)	9.1 (8.9–9.2)	1.98 (1.91–2.06)	1.56 (1.50–1.62)
**GDP level**						
Tertitle 1	32.2 (32.1–32.3)	1.00	1.00	5.9 (5.8–6.0)	1.00	1.00
Tertitle 2	33.4 (33.2–33.5)	1.05 (1.04–1.06)	0.94 (0.93–0.95)	5.8 (5.7–5.9)	0.99 (0.98–1.01)	0.89 (0.88–0.91)
Tertitle 3	35.8 (35.7–35.9)	1.17 (1.16–1.18)	1.11 (1.10–1.12)	7.3 (7.3–7.4)	1.28 (1.26–1.29)	1.20 (1.18–1.21)

OR, odds ratio; CI, confidence interval. ^a^Data are shown as prevalence (95% confidence interval), and all estimates were weighted using the China Population Census in 2010. Overweight and obesity were defined according to the Chinese criteria: overweight, 24.0 ≤ BMI ≤ 27.9 kg/m^2^ and obesity, BMI ≥ 28.0 kg/m^2^. ^b^Adjusted for all other factors in the table.

**Figure 1:**
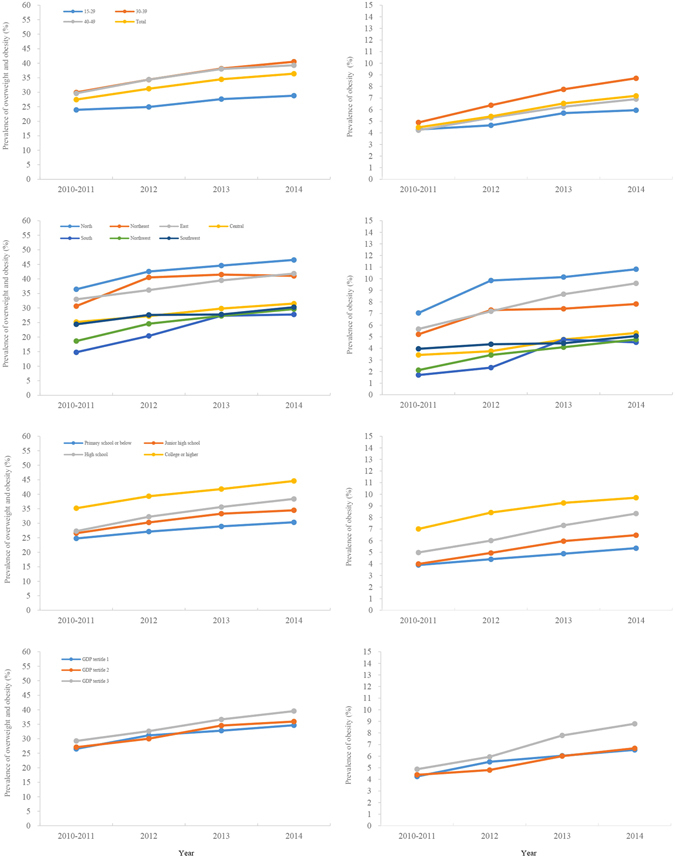
Prevalence and overweight and obesity among men aged 15–49 years by demographic variables, 2010–2014. Overweight and obesity were defined according to the Chinese criteria: overweight, 24.0 ≤ BMI ≤ 27.9 kg/m^2^ and obesity, BMI ≥ 28.0 kg/m^2^.

**Figure 2:**
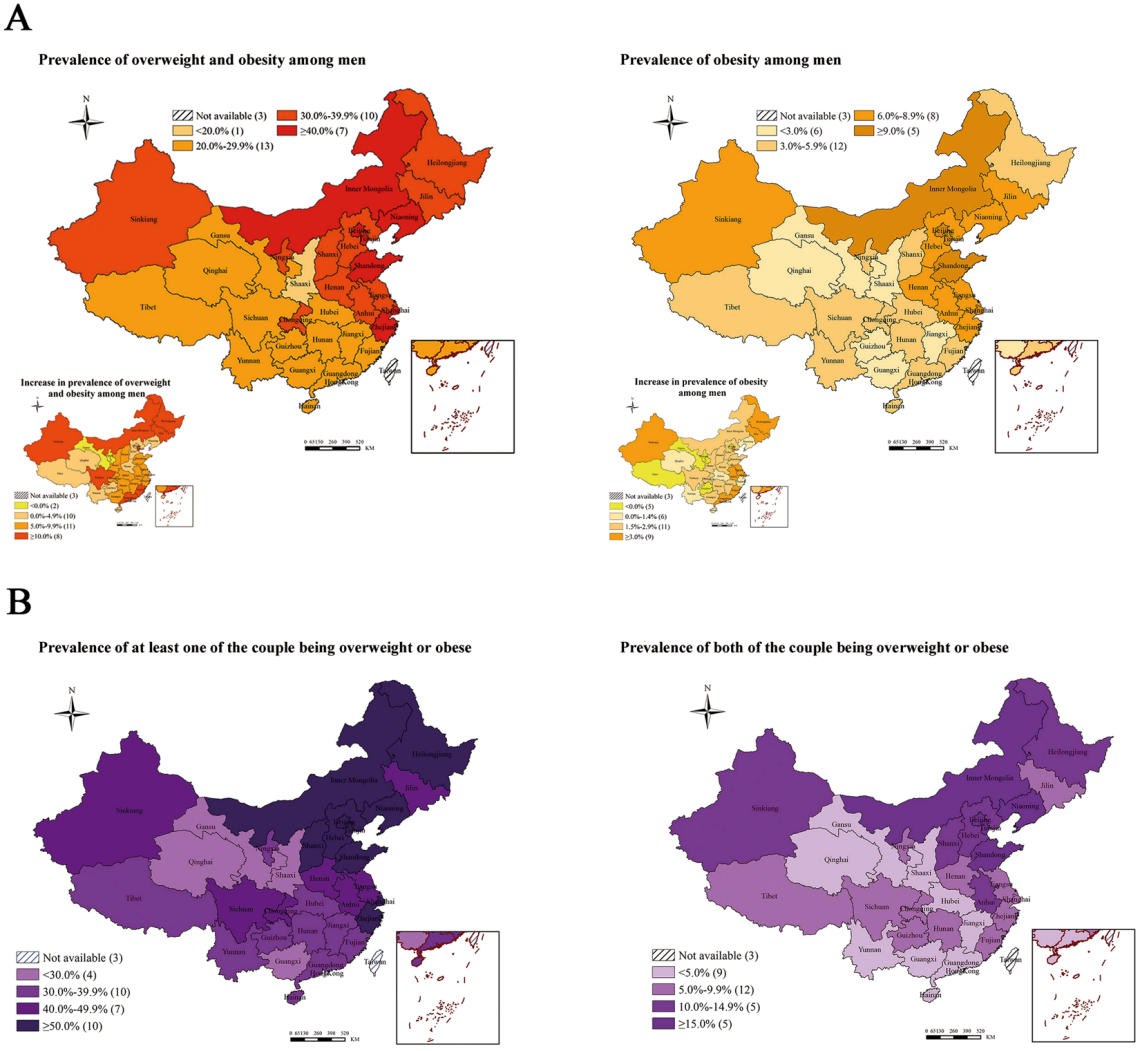
(**A**) Prevalence and trends in overweight and obesity among reproductive-age men in 31 provinces, municipalities and autonomous regions, 2010–2014; (**B**) Clustering of overweight and obesity among couples in 31 provinces, municipalities and autonomous regions, 2010–2014. Overweight and obesity were defined according to Chinese criteria: overweight, 24.0 ≤ BMI ≤ 27.9 kg/m^2^ and obesity, BMI ≥ 28.0 kg/m^2^; Couples: those with at least one of them being overweight or obese. Figure 2 is created by using the software ArcGIS 10.2 (http://www.esri.com/software/arcgis).

During the study period, the overall prevalence increased by 8.9% (95% CI 8.6–9.2%) for combined overweight and obesity, and 2.7% (95% CI 2.6–2.9%) for obesity alone defined by the Chinese criteria (all P < 0.0001; Fig. 1 and Table 3). The increase was present in almost all subgroups, although substantial disparities were observed (Table 3). Greater increases in overweight and obesity were observed in men aged 30 years or above, with Han background, with higher education, or in the high GDP region. In addition, the increases were significantly faster in regions of north, northeast, south and northwest. According to the WHO criteria, the prevalence increased by 7.6% (95% CI 7.3–7.8%) for combined overweight and obesity, and 1.1% (95% CI 1.0–1.2%) for obesity alone (See Supplementary Table S2). Information on the prevalence and trend of overweight and obesity in 31 provinces, municipalities and autonomous regions during 2010 and 2014 is shown in Fig. 2A. Again, substantial variations were observed across different regions in China.

**Table 3:** Trends in prevalence of overweight and obesity among reproductive-age men aged 15–49 years in rural China, 2010–2014^a^.

	Overweight and obesity				Obesity			
	2010–2011	2014	Change 2010–2011 to 2014, point (95% CI)	p value	2010–2011	2014	Change 2010–2011 to 2014, point (95% CI)	p value
**Total**	27.5 (27.2–27.7)	36.4 (36.2–36.5)	8.9 (8.6–9.2)	<0.0001	4.5 (4.3–4.6)	7.2 (7.1–7.3)	2.7 (2.6–2.9)	<0.0001
**Age groups**								
15–29	23.9 (23.6–24.2)	28.8 (28.7–28.9)	4.9 (4.5–5.2)	<0.0001	4.3 (4.1–4.5)	6.0 (5.9–6.1)	1.7 (1.5–1.8)	<0.0001
30–39	29.9 (29.6–30.2)	40.5 (40.4–40.7)	10.6 (10.3–10.9)	<0.0001	4.9 (4.7–5.1)	8.7 (8.6–8.8)	3.8 (3.6–4.0)	<0.0001
40–49	29.6 (28.8–30.4)	39.3 (39.0–39.7)	9.7 (8.8–10.5)	<0.0001	4.2 (3.9–4.6)	6.9 (6.7–7.1)	2.7 (2.3–3.1)	<0.0001
**Ethnic origin**								
Han	27.4 (27.1–27.7)	37.1 (37.0–37.3)	8.3 (8.0–8.5)	<0.0001	4.5 (4.4–4.6)	7.4 (7.3–7.5)	1.2 (1.1–1.3)	<0.0001
Uyghur	29.1 (28.0–30.3)	35.6 (35.0–36.3)	3.7 (2.6–4.9)	<0.0001	4.7 (4.2–5.3)	6.0 (5.6–6.4)	0.5 (0.3–0.7)	<0.0001
Zhuang	15.0 (13.8–16.3)	22.9 (22.3–23.6)	7.7 (6.9–8.6)	<0.0001	1.1 (0.8–1.3)	2.8 (2.6–3.1)	0.5 (0.3–0.7)	<0.0001
Miao	19.8 (18.1–21.7)	26.6 (25.7–27.5)	3.6 (1.9–5.3)	<0.0001	3.3 (2.5–4.3)	3.7 (3.3–4.1)	0.2 (−0.3–0.7)	0.376
Manchu	39.4 (37.0–41.7)	43.4 (41.9–44.9)	5.0 (2.6–7.4)	<0.0001	5.8 (4.8–7.0)	9.6 (8.8–10.6)	1.5 (0.6–2.3)	0.001
Mongol	35.9 (33.1–38.8)	45.1 (43.8–46.4)	6.4 (3.4–9.4)	<0.0001	8.7 (6.8–11.0)	10.1 (9.4–10.8)	0.7 (−0.9–2.3)	0.395
Yi	19.8 (18.3–21.3)	25.0 (23.9–26.1)	3.5 (2.0–5.2)	<0.0001	2.5 (2.1–2.9)	4.7 (4.2–5.3)	0.4 (0–0.8)	0.045
Hui	37.0 (30.2–44.2)	28.1 (27.2–29.0)	−5.1 (−1.3–2.3)	0.176	6.6 (5.1–8.6)	6.2 (5.7–6.7)	−0.2 (−1.2–0.8)	0.671
Tujia	26.3 (23.2–30.0)	27.9 (26.7–29.0)	2.1 (−0.7–4.8)	0.136	3.9 (2.6–5.8)	3.7 (3.3–4.1)	0.2 (−0.6–1.1)	0.579
Tibetan	14.8 (11.8–18.4)	25.3 (23.5–27.1)	6.8 (3.7–9.8)	<0.0001	1.5 (0.9–2.8)	4.2 (3.2–5.5)	0.8 (0–1.5)	0.054
**Region**								
North	36.4 (35.4–37.5)	46.5 (46.0–47.0)	10.1 (8.9–11.2)	<0.0001	7.0 (6.5–7.6)	10.8 (10.5–11.1)	3.8 (3.2–4.4)	<0.0001
Northeast	30.6 (29.7–31.6)	41.0 (40.5–41.5)	10.4 (9.3–11.5)	<0.0001	5.2 (4.8–5.7)	7.8 (7.6–8.1)	2.6 (2.1–3.1)	<0.0001
East	33.0 (32.4–33.6)	41.8 (41.5–42.2)	8.8 (8.2–9.5)	<0.0001	5.7 (5.4–5.9)	9.6 (9.4–9.8)	3.9 (3.6–4.3)	<0.0001
Central	25.2 (24.9–25.5)	31.5(31.3–31.7)	6.3 (5.9–6.7)	<0.0001	3.4 (3.3–3.6)	5.3 (5.2–5.4)	1.9 (1.7–2.1)	<0.0001
South	14.8 (14.4–15.2)	27.8 (27.5–28.0)	13.0 (12.5–13.5)	<0.0001	1.7 (1.6–1.9)	4.5 (4.4–4.6)	2.8 (2.6–3.0)	<0.0001
Northwest	18.6 (18.2–19.1)	29.6 (29.3–30.0)	11.0 (10.4–11.6)	<0.0001	2.1 (2.0–2.3)	4.8 (4.6–4.9)	2.6 (2.4–2.9)	<0.0001
Southwest	24.4 (23.9–24.9)	30.3 (30.0–30.6)	5.9 (5.3–6.5)	<0.0001	4.0 (3.7–4.2)	5.1 (4.9–5.2)	1.1 (0.8–1.4)	<0.0001
**Education**								
Primary school or below	24.8 (23.8–25.7)	30.3 (29.8–30.8)	5.6 (4.5–6.6)	<0.0001	3.9 (3.4–4.4)	5.4 (5.1–5.6)	1.4 (0.9–2.0)	<0.0001
Junior high school	26.6 (26.2–26.9)	34.5 (34.3–34.6)	7.9 (7.5–8.3)	<0.0001	4.0 (3.8–4.1)	6.4 (6.3–6.6)	2.5 (2.3–2.7)	<0.0001
High school	27.2 (26.6–27.9)	38.4 (38.0–38.8)	11.1 (10.4–11.9)	<0.0001	5.0 (4.7–5.3)	8.3 (8.1–8.5)	3.4 (3.0–3.7)	<0.0001
College or higher	35.2(34.3–36.1)	46.1 (45.7–46.5)	9.4 (8.4–10.4)	<0.0001	7.0 (6.5–7.5)	9.7 (9.4–10.0)	2.7 (2.1–3.2)	<0.0001
**GDP level**								
Tertitle 1	26.5 (26.1–27.0)	34.7 (34.4–34.9)	8.1 (7.6–8.7)	<0.0001	4.3 (4.0–4.5)	6.5 (6.4–6.7)	2.3 (2.0–2.5)	<0.0001
Tertitle 2	27.1 (26.6–27.6)	35.9 (35.6–36.2)	8.9 (8.3–9.4)	<0.0001	4.4 (4.2–4.6)	6.7 (6.5–6.9)	2.3 (2.0–2.5)	<0.0001
Tertitle 3	29.2 (28.8–29.7)	39.5 (39.3–39.8)	10.3 (9.8–10.8)	<0.0001	4.9 (4.7–5.1)	8.8 (8.7–8.9)	3.9 (3.7–4.1)	<0.0001

^a^Data are shown as prevalence (95% confidence interval), and all estimates were weighted using the China Population Census in 2010. The sample size in 2010 was relatively small, thus data from 2010 and 2011 were combined. Overweight and obesity were defined according to the Chinese criteria: overweight, 24.0 ≤ BMI ≤ 27.9 kg/m^2^ and obesity, BMI ≥ 28.0 kg/m^2^.

Data from 15 997 739 men and their female partners could be matched in this study. Among them, increasing trends in prevalence of overweight and obesity were observed in both men and women (See Supplementary Fig. S1). During the study years, the proportion of couples with at least one of them being overweight or obese was 45.8%, whereas proportion of couples with both of them being overweight or obesity was 9.9% based on the Chinese criteria (Table 4). The clustering of overweight and/or obesity in couples was stronger in older age groups, particularly in women, and in ethnic groups of Uyghur and Mongolian. Higher education was positively associated with higher prevalence of overweight or obesity in couples, whereas it was inversely associated with simultaneous overweight and obesity in men and women. Positive associations between higher GDP level and couples’ overweight and/or obesity were consistent. Similarly, the prevalence of the clustering overweight/obesity was lower in the south region, and higher in the north region (Table 4). In addition, the prevalence of overweight and/or obesity in couples in 31 provinces, municipalities and autonomous regions is also presented in Fig. 2B.

**Table 4:** Clustering of overweight and obesity for reproductive-age couples in rural China, 2010–2014^a^.

	At least one of the couple being overweight or obese			Both of the couple being overweight or obese		
	Prevalence	Crude OR (95%CI)	Adjusted OR (95%CI) ^b^	Prevalence	Crude OR (95%CI)	Adjusted OR (95%CI)^b^
**Total**	45.8 (45.7–45.9)			9.9 (9.8–10.0)		
**Male age groups**						
15–29	34.0 (33.8–34.2)	1.00	1.00	4.9 (4.8–5.0)	1.00	1.00
30–39	51.2 (51.1–51.4)	2.04 (2.02–2.06)	1.54 (1.52–1.57)	11.5 (11.4–11.6)	2.54 (2.48–2.59)	1.75 (1.70–1.80)
40–49	55.6 (55.3–55.8)	2.43 (2.40–2.47)	1.50 (1.47–1.54)	14.9 (14.7–15.2)	3.42 (3.34–3.51)	1.74 (1.67–1.82)
**Female age groups**						
15–29	34.4 (34.2–34.6)	1.00	1.00	4.9 (4.8–5.0)	1.00	1.00
30–39	52.5 (52.4–52.6)	2.11 (2.09–2.13)	1.67 (1.65–1.69)	12.1 (12.0–12.2)	2.65 (2.60–2.70)	2.04 (1.99–2.09)
40–49	56.1 (55.8–56.3)	2.43 (2.39–2.47)	1.79 (1.75–1.84)	15.3 (15.1–15.5)	3.49 (3.40–3.57)	2.36 (2.27–2.46)
**Male ethnic origin**						
Han	46.8 (46.6–46.9)	1.00	1.00	10.1 (10.0–10.2)	1.00	1.00
Uyghur	40.3 (39.6–41.0)	0.77 (0.75–0.79)	1.54 (1.50–1.57)	9.9 (9.5–10.3)	0.97 (0.92–1.01)	2.22 (2.13–2.32)
Zhuang	28.3 (27.6–29.1)	0.45 (0.43–0.47)	0.58 (0.56–0.60)	3.8 (3.6–4.1)	0.35 (0.33–0.37)	0.50 (0.47–0.54)
Miao	30.0 (28.3–31.6)	0.49 (0.45–0.52)	0.93 (0.89–0.97)	4.5 (4.1–4.8)	0.41 (0.38–0.45)	0.93 (0.86–1.02)
Manchu	61.8 (60.7–62.9)	1.84 (1.76–1.93)	1.11 (1.05–1.17)	16.8 (15.9–17.7)	1.79 (1.68–1.90)	0.99 (0.93–1.05)
Mongol	61.0 (59.8–62.1)	1.78 (1.69–1.86)	1.38 (1.31–1.44)	17.5 (16.7–18.3)	1.88 (1.77–1.99)	1.40 (1.32–1.49)
Yi	33.0 (31.2–34.9)	0.56 (0.52–0.61)	1.04 (0.98–1.12)	4.6 (4.1–5.2)	0.43 (0.38–0.48)	0.92 (0.81–1.05)
Hui	29.3 (28.3–30.2)	0.47 (0.45–0.49)	1.19 (1.14–1.24)	4.8 (4.5–5.3)	0.45 (0.42–0.49)	1.42 (1.32–1.52)
Tujia	40.6 (39.5–41.6)	0.78 (0.78–0.81)	0.83 (0.79–0.87)	7.0 (6.5–7.6)	0.67 (0.62–0.73)	0.73 (0.67–0.80)
Tibetan	32.1 (30.0–34.3)	0.54 (0.49–0.59)	0.93 (0.85–1.02)	7.2 (6.2–8.4)	0.69 (0.58–0.82)	1.25 (1.05–1.50)
**Female ethnic origin**						
Han	46.8 (46.6–46.9)	1.00	1.00	10.1 (10.0–10.2)	1.00	1.00
Uyghur	40.4 (39.6–41.1)	0.77 (0.75–0.79)	1.50 (1.41–1.59)	9.9 (9.5–10.3)	0.97 (0.93–1.02)	2.21 (2.01–2.42)
Zhuang	28.9 (28.2–29.6)	0.46 (0.45–0.48)	0.62 (0.59–0.65)	3.8 (3.6–4.1)	0.35 (0.33–0.37)	0.51 (0.46–0.55)
Miao	30.2 (28.7–31.8)	0.49 (0.46–0.53)	0.89 (0.82–0.96)	4.7 (4.3–5.1)	0.44 (0.40–0.48)	0.85 (0.76–0.94)
Manchu	62.3 (61.2–63.4)	1.88 (1.79–1.97)	1.17 (1.11–1.24)	17.4 (16.4–18.3)	1.86 (1.74–1.99)	1.07 (1.00–1.16)
Mongol	61.5 (60.4–62.6)	1.82 (1.74–1.90)	1.38 (1.31–1.46)	17.8 (16.9–18.7)	1.92 (1.81–2.04)	1.43 (1.33–1.54)
Yi	33.9 (32.2–35.7)	0.58 (0.54–0.63)	1.09 (1.00–1.18)	5.1 (4.5–5.7)	0.47 (0.42–0.53)	0.96 (0.85–1.09)
Hui	29.8 (28.8–30.8)	0.48 (0.46–0.51)	0.89 (0.85–0.94)	5.0 (4.6–5.3)	0.46 (0.43–0.50)	0.98 (0.90–1.06)
Tujia	41.1 (40.0–42.2)	0.79 (0.76–0.83)	0.87 (0.83–0.92)	7.1 (6.4–8.5)	0.68 (0.62–0.74)	0.75 (0.68–0.82)
Tibetan	32.1 (30.1–34.2)	0.54 (0.49–0.59)	0.91 (0.83–1.00)	7.4 (6.4–8.5)	0.70 (0.60–0.82)	1.21 (1.03–1.42)
**Region**						
North	58.5 (58.1–58.9)	3.05 (2.99–3.11)	3.78 (3.69–3.85)	15.2 (14.9–15.4)	3.78 (3.66–3.91)	4.68 (4.52–4.85)
Northeast	56.9 (56.5–57.3)	2.86 (2.81–2.92)	2.78 (2.71–2.83)	15.0 (14.7–15.3)	3.73 (3.60–3.87)	3.31 (3.19–3.44)
East	51.9 (51.6–52.2)	2.34 (2.31–2.37)	2.37 (2.34–2.41)	11.7 (11.5–11.9)	2.81 (2.72–2.90)	2.80 (2.72–2.89)
Central	40.6 (40.4–40.8)	1.48 (1.46–1.50)	1.52 (1.50–1.54)	7.2 (7.1–7.3)	1.65 (1.60–1.70)	1.64 (1.59–1.69)
South	31.6 (31.3–31.8)	1.00	1.00	4.5 (4.4–4.6)	1.00	1.00
Northwest	32.1 (31.7–32.4)	1.02 (1.01–1.04)	1.54 (1.51–1.57)	5.9 (5.7–6.0)	1.33 (1.28–1.38)	2.05 (1.97–2.14)
Southwest	40.8 (40.4–41.1)	1.49 (1.47–1.52)	1.78 (1.74–1.81)	7.8 (7.6–7.9)	1.78 (1.72–1.85)	2.01 (1.93–2.09)
**Male education**						
Primary school or below	42.3 (41.9–42.8)	1.00	1.00	9.5 (9.3–9.8)	1.00	1.00
Junior high school	45.9 (45.7–46.0)	1.16 (1.13–1.18)	0.99 (0.96–1.01)	10.4 (10.3–10.5)	1.10 (1.06–1.13)	0.96 (0.93–1.00)
High school	44.7 (44.3–45.0)	1.10 (1.07–1.13)	1.02 (0.99–1.05)	9.1 (8.9–9.3)	0.95 (0.92–0.99)	0.98 (0.94–1.03)
College or higher	50.0 (49.7–50.3)	1.36 (1.33–1.39)	1.21 (1.17–1.26)	8.6 (8.4–8.8)	0.89 (0.86–0.92)	0.95 (0.89–1.00)
**Female education**						
Primary school or below	43.8 (43.3–44.3)	1.00	1.00	10.0 (9.8–10.3)	1.00	1.00
Junior high school	45.7 (45.5–45.8)	1.08 (1.06–1.10)	0.95 (0.93–0.97)	10.3 (10.2–10.4)	1.03 (0.99–1.06)	0.94 (0.91–0.97)
High school	44.6 (44.3–44.9)	1.03 (1.01–1.06)	0.96 (0.93–0.99)	9.0 (8.8–9.2)	0.89 (0.86–0.92)	0.94 (0.90–0.99)
College or higher	50.0 (49.7–50.3)	1.28 (1.26–1.31)	1.10 (1.07–1.14)	8.6 (8.4–8.7)	0.84 (0.81–0.87)	0.88 (0.84–0.94)
**GDP level**						
Tertitle 1	43.5 (43.3–43.7)	1.00	1.00	9.4 (9.3–9.5)	1.00	1.00
Tertitle 2	47.7 (47.4–47.9)	1.18 (1.17–1.20)	0.99 (0.97–1.00)	10.6 (10.4–10.7)	1.14 (1.11–1.17)	0.94 (0.92–0.96)
Tertitle 3	47.6 (47.4–47.8)	1.18 (1.17–1.19)	1.14 (1.12–1.15)	10.3 (10.1–10.4)	1.10 (1.08–1.13)	1.19 (1.17–1.22)

OR, odds ratio; CI, confidence interval. ^a^Data are shown as prevalence (95% confidence interval), and all estimates were weighted using the China Population Census in 2010. Overweight and obesity were defined according to the Chinese criteria: overweight, 24.0 ≤ BMI ≤ 27.9 kg/m^2^ and obesity, BMI ≥ 28.0 kg/m^2^. ^b^Adjusted for all other factors in the table.

## Discussion

In this largest study to date in rural China, about one third of reproductive-age men aged 15–49 years were overweight/obese and 6.3% of them were obese according to the Chinese criteria. By examining patterns of reginal difference, overweight and obesity prevalence was substantial high in North China, and was low in the south or northwest regions. Of particular relevance is the ethnicity and cultural differences in rural China. The increase trend of overweight and obesity was common in young men, especially among those in higher socioeconomic strata. These findings indicate that the epidemic of overweight and obesity has become a serious public problem among reproductive-age men in rural China.

The weight status in Chinese populations have been examined in general population by a few national surveys. The China National Nutrition and Health Survey (CNNHS) reported that the prevalence of overweight and obesity (BMI ≥ 24.0 kg/m^2^) in men aged 18–44 years rose rapidly from 14.5% to 30.4% between 1992 and 2002^12^. The annual increasing rate was much higher in younger men aged 18–44 years (1.6% per year) compared with men aged 45–59 years (1.0% per year) and those aged 60 years or above (0.6% per year). A series of China Health and Nutrition Survey (CHNS) studies found that the proportions of men with a BMI ≥ 25 kg/m^2^ has tripled from 8.2% to 26.5% in the age group of 18–39 years between 1993 and 2009, and the increase was larger than other age groups or in women^11^. In Western countries, the prevalence of overweight/obesity remained high in young men of reproductive age: 62% in US^10^, 56.1% in Canada^13^, and 42% in England^14^. Comparing with these findings, our results (33.8% according to the Chinese criteria and 22.4% according to the WHO criteria) were lower than that in Western countries, but were generally consistent with results from other smaller studies in Chinese populations.

In the analysis of different geographical regions, the north and northeast were associated with high prevalence of overweight and obesity in rural China. Some provinces even had a high prevalence close to that in the US (two thirds of the total young men)^10^. Among our participants, several minority ethnic groups (eg. 43.7% for Mongolian and 43.0% for Manchu) have substantially higher rates of overweight and obesity than do Han ethnic group (34.0%). These reginal/ethnic variations could be attributed to the socioeconomic status and the accompanying lifestyle/culture influence, as well as different genetic backgrounds^3^. People from rural regions are experiencing the nutrition transition towards a high-fat, high-energy diet, and sedentary lifestyles^15^. Particularly for some minority ethnic groups, such as Mongolian and Manchu, those who mainly live in regions of north and northeast maintain a traditional meat dietary pattern for the reasons of favoring a large body and need against the cold^16^. Previous evidence also suggests that people living in the north, rural areas consumed more alcohol beverage than their counterparts, and some minority ethnic groups, such as those of Mongolian background, had higher levels of alcohol consumption than other ethnic groups^17^. Additionally, socioeconomic status (eg. personal educational level, area-level economic status) impacts the prevalence of overweight and obesity that men of higher social strata are more likely to be obese in developing countries^18^. In our study, the conclusions were consistent that overweight/obesity prevalence was relatively higher among rural men with higher level of education or living in the high GDP areas. Furthermore, there is a clear distinction regarding to genetic predisposition on obesity in different Chinese populations; the characteristics of gut microbiota in Han population are different from minority ethnic groups, which plays an important role in obesity epidemic^19, 20^. In short, rates of overweight/obesity have increased among rural people of all ages and regions, but some groups are disproportionately affected. Along with the rapid social and economic change with increasing urbanization, the growing burden of overweight and obesity will affect more reproductive-age men across China in the near future.

Both paternal and maternal obesity are related to the health of the next generation^5^. Maternal prepregnancy overweight/obesity was associated with adverse pregnancy outcomes^21, 22^. Male obesity also negatively impacts fertility by changes in the hormones, reduced semen functions, as well as changes in sperm epigenetic marks^23^. Therefore, it is important to assess the clustering of overweight and obesity couples. In our study, nearly half of rural couples had at least one of them being overweight or obese before conception, and higher prevalence was associated with increasing age, higher socioeconomic strata, specific ethnicity and regions. Meanwhile, about 10% of the couples were overweight or obesity in both of them, and those couples should be prioritized for the interventions. Obesity intervention prior to conception offers unique opportunity for improving pregnancy outcomes, offspring health and also parental health. Overweight/obese couples may share common factors including diet, physical activities and health behaviors, and intervention targeting both couples has been proved more effective than targeting men or women alone^24, 25^. There is an urgent need for more studies on effective and cost-effective methods of preconception care with an emphasize on weight management, which has been overlooked to a great extent in our society.

Our study has several strengths. First, we included more than 15 million rural men aged 15–49 years in this mega national survey. The study sample covered 80% of the target population in the rural China for couples who intended to get pregnant soon. In addition, we have used a two-stage stratified cluster sampling method and standardized the results to the 2010 China Population Census. Therefore, we considered the results to be representative of actual prevalence of overweight and obesity in reproductive-age men in rural China. Moreover, we utilized the data from couples to examine the distribution of both spouse’s BMI in a general healthy population planning pregnancy. Few studies have analyzed the BMI distribution of a family, while this is certainly important for pregnancy and health of the next generation. We also acknowledge the following limitations of our study. First, our findings are based on a non-random sampling method of 31 provinces, municipalities and autonomous regions, thus the generalization to the entire male population of 15–49 years old in rural China should be cautious. However, due to the very high coverage proportion, we did not think that the results will be materially changed if random sample or total population approaches were used. Second, the majority of men in this study were from rural areas, and the true prevalence of overweight and obesity could be underestimated in the total population because the overweight/obesity prevalence seemed to be slightly higher in urban areas than rural areas in China^12, 26, 27^. Finally, between-investigator variations in the measurement of body weight and height were possible. However, all study staff (doctors, nurses, and health workers) were adequately trained in standard protocol and the measurement errors could be random, if exist. Given the large sample size, it is unlikely that measure errors could have substantially altered the results.

In conclusion, our study found that about one-third of Chinese young men of reproductive age in rural areas were overweight or obese, and we also observed an increasing trend in the prevalence across the study years. Substantial disparities across geographic regions were observed in the prevalence of overweight and obesity. Further studies are needed to explore the reasons for the disparities, and region-specific health resources and public policies should also be explored to reduce the disease burden of overweight and obesity. In addition, about 45% of couples had at least one of them being overweight or obesity, which will have an enormous impact on the population health of the adults themselves and also the health for the next generation in China. Therefore, effective weight loss strategies are needed for the couples who plan to get pregnant in order to reduce pregnancy complications and improve overall population health.

## Methods

### Study design and participants

The NFPHEP, launched by National Health and Family Planning Commission and Finance Ministry, is an ongoing preconception care service for rural couples of reproductive age planning pregnancy across China since 2010. The couples who were planning pregnancy within the next 6 months, at least one of whom was defined as a rural resident were eligible for participating in the project. Participants received preconception care including free health checkups, risk assessments and health consultations, as well as two follow-ups for pregnancy outcomes, with the purpose of reducing the potential risk factors for birth defects and improving population health. The NFPHEP began with 100 rural counties in 2010, and extended to 220 rural counties in 2011 and were further extended to 2790 counties of 31 provinces in 2013 afterwards. Before 2013, the numbers of NFPHEP unit were selected in proportion to the local population size and numbers of total counties in each province with a two-stage stratified cluster sampling method. Since NFPHEP is a free healthcare program aiming to improve pregnancy outcomes and reduce birth defects, and women voluntarily came to get the services. Details of the study design, process and data collection are published elsewhere^28^. In our study, a total of 17 269 054 men who participated in the program between 2010 and 2014 were included in this study. We excluded 289 049 participants aged beyond 20–49 years, 818 023 without physical examination information on weight and height. Finally, data from 16 161 982 of the participants aged between 15 and 49 years were used for statistical analyses. Also we included 15 997 739 female partners, who aged 15–49 years and had complete height and weight information. The study was conducted in accordance with the Declaration of Helsinki and approved by the ethics committees of the National Health and Family Planning Commission and National Research Institute for Family Planning. Informed consent in Chinese was obtained from all participants before any data collection.

### Data Collection

Data from 31 provinces, municipalities and autonomous regions between 2010 and 2014 were included. During the physical examination, weight and height were measured after participants had removed shoes and heavy clothes. Height (measured to nearest 0.1 cm) and weight (measured to the nearest 0.1 kg) were measured by trained personnel using standardized equipment and protocol provided by the National Research Institute for Family Planning. Body mass index (BMI) was calculated as weight in kilograms divided by height in meters squared and rounded to the nearest tenth. All participants also filled in a standard questionnaire to provide basic demographic information including age, education level and residence address through a face-to-face interview by trained local health staff.

The participants with missing information on height or weight or extreme or implausible BMI values were all excluded in this study. Overweight and obesity were defined according to the Chinese definitions [overweight: BMI 24.0–27.9 kg/m^2^; obesity: BMI ≥ 28.0 kg/m^2^] and WHO definitions [overweight: BMI 25.0–29.9 kg/m^2^; obesity: BMI ≥ 30.0 kg/m^2^], respectively^29, 30^. Individuals were grouped by age: 15–29, 30–39 and 40–49 years. The geographical locations were classified as North China (Beijing, Hebei, Inner Mongolia, Shanxi, Tianjin), Northeast China (Heilongjiang, Jilin, Liaoning), East China (Anhui, Fujian, Jiangsu, Jiangxi, Shandong, Shanghai, Zhejiang), Central China (Henan, Hubei, Hunan), South China (Guangdong, Guangxi, Hainan), Northwest China (Gansu, Ningxia, Qianghai, Shaanxi, Sinkiang) and Southwest China (Guizhou, Sichuan, Tibet, Yunnan, Chongqing). Education levels were divided into four groups of primary school or lower, junior high school, high school, and college or above. Economic development was divided into tertiles of gross domestic product (GDP) per head in 2013 with the following ranges: 0.2–3.4, 3.5–4.3, and 4.4–10.0 (CNY10 000), respectively.

### Statistical Analysis

All analyses took into account the deviations in the NFPHEP sample compared with the standard population of 2010 China Population Census, using the weight coefficients adjusted for age for every one year and geographical region. Standard errors were estimated with Taylor series linearization method. The adjusted prevalence of overweight and obesity and 95% confidence intervals (CIs) were reported in total and by age, region, education and GDP subgroups. Differences within sociodemographic subgroups were tested by χ² test. Then, we analyzed the trends and absolute changes in prevalence of overweight and obesity from 2010 to 2014 using t statistics and orthogonal contrast matrices, and reported the p value for trends. We further reported the clustering of overweight/obesity among couples, defined as at least one of them was classified as overweight or obesity, or both of them were overweight or obesity. In addition, multivariable logistic regressions were used to explore the association between relevant covariates (age, geographic location, education level and GDP level) and overweight/obesity, and the crude and adjusted odds ratios (ORs) and 95% CIs were reported. Statistical inference was based on 2-sided test and p value < 0.05. All data analyses involved use of STATA software version 13.0.

## Electronic supplementary material

Supplemental Materials
