# Supplementary material for: Prevalence of overweight and obesity in 15.8 million men aged 15–49 years in rural China from 2010 to 2014
Source: Sci Rep. 2017 Jul 10;7:5012. doi: 10.1038/s41598-017-04135-4 (PMC5504069; doi:10.1038/s41598-017-04135-4)
Supplement: Supplementary file 1 — Supplemental Materials [file 41598_2017_4135_MOESM1_ESM.pdf]

Prevalence of overweight and obesity in 15.8 million men aged 15-49 years in rural China: a population-based, cross-sectional study from 2010 to 2014

Yuan He, An Pan, Yuanyuan Wang, Ying Yang, Jihong Xu, Ya Zhang, Dujia Liu, Qiaomei Wang, Haiping Shen, Yiping Zhang, Donghai Yan, Zuoqi Peng, Frank B. Hu, Xu Ma

Online Supplemental Material

The following materials are included in the Online Supplemental Material.

1. Supplemental Table S1.
2. Supplemental Table S2.
3. Supplemental Figure S1.

Supplemental Table S1. Sample sizes and weighted proportion of demographic characteristics of women in rural China between 2010 and 2014.
Supplemental Table S2. Prevalence and trends of overweight and obesity according to the WHO criteria for reproductive-age men aged 15-49 years in rural China, 2010-2014
Supplemental Figure S1. Trends in prevalence of overweight and obesity among men and their female partners before conception, 2010-2014.
Figure legends: Overweight and obesity were defined according to the Chinese criteria: overweight, 24.0≤ BMI ≤27.9 kg/m^2 and obesity, BMI ≥28.0 kg/m^2; Couples: those with at least one of them being overweight or obese.

Supplemental Table S1. Sample sizes and weighted proportion of demographic characteristics of reproductive women in rural China between 2010 and 2014. [a]

	N	percentage
**Total**	15 997 739	100%
**Age groups**		
15-29	12 450 050	77.8
30-39	3 206 772	20.1
40-49	340 917	2.14
**Ethnic origin**		
Han	13 960 474	90.6
Uyghur	360 889	2.3
Zhuang	281 826	1.8
Miao	163 347	1.1
Manchu	93 316	0.6
Mongol	88 073	0.6
Yi	90 461	0.6
Hui	170 795	1.1
Tujia	125 601	0.8
Tibetan	67 590	0.5
**Region**		
North	1 355 720	8.5
Northeast	643 822	4.0
East	4 272 896	26.7
Central	4 096 408	25.6
South	2 226 543	13.9
Northwest	1 397 510	8.7
Southwest	2 005 840	12.4
**Education**		
Primary school or below	968 252	6.2
Junior high school	9 762 112	62.4
High school	2 839 046	18.2
College or higher	2 063 777	13.2
**GDP level**		
Tertitle 1	5 932 661	37.1
Tertitle 2	4 851 831	30.3
Tertitle 3	5 213 247	32.6

[a]The data are shown as percentage of participants in each year, and all proportion estimates were weighted using the China Population Census in 2010.

Supplemental Table S2. Prevalence and trends of overweight and obesity according to the WHO criteria for reproductive-age men aged 15-49 years in rural China, 2010-2014 [a]

	Overweight and obesity		Obesity	
	Prevalence	Increase	Prevalence	Increase
**Total**	22.4 (22.3-22.5)	7.6 (7.3-7.8)	2.5 (2.4-2.6)	1.1 (1.0-1.2)
**Age groups**				
15-29	17.9 (17.8-18.0)	4.2 (3.9-4.5)	2.3 (2.2-2.4)	0.8 (0.7-0.9)
30-39	25.5 (25.4-25.5)	9.2 (8.9-9.6)	3.0 (2.9-3.1)	1.7 (1.6-1.8)
40-49	24.0 (23.8-24.2)	8.6 (7.9-9.3)	2.2 (2.1-2.3)	0.9 (0.7-1.2)
**Ethnic origin**				
Han	22.8 (22.7-22.9)	8.3 (8.0-8.5)	2.6 (2.5-2.6)	1.2 (1.1-1.3)
Uyghur	20.9 (20.6-21.2)	3.7 (2.6-4.9)	2.2 (2.1-2.4)	0.5 (0.1-1.0)
Zhuang	13.0 (12.7-13.3)	7.7 (6.9-8.6)	0.9 (0.8-1.0)	0.5 (0.3-0.7)
Miao	14.6 (14.2-15.0)	3.6 (1.9-5.3)	1.2 (1.0-1.3)	0.2 (-0.3-0.7)
Manchu	28.6 (27.9-29.3)	5.0 (2.6-7.4)	3.6 (3.4-3.9)	1.5 (0.6-2.3)
Mongol	29.5 (28.8-30.1)	6.4 (3.4-9.4)	4.4 (4.1-4.6)	0.7 (-0.9-2.3)
Yi	15.0 (14.4-15.6)	3.6 (2.0-5.2)	1.4 (1.2-1.6)	0.4 (0-0.8)
Hui	19.3 (18.8-19.9)	-5.1 (-1.25-2.29)	2.6 (2.3-2.9)	-0.2 (-1.2-0.8)
Tujia	15.6 (15.1-16.1)	2.1 (-0.7-4.8)	1.1 (1.0-1.3)	0.2 (-0.6-1.1)
Tibetan	13.8 (12.9-14.7)	6.8 (3.7-9.8)	1.3 (1.0-1.8)	0.8 (0-1.5)
**Region**				
North	30.3 (30.0-30.6)	10.4 (9.4-11.4)	4.4 (4.3-4.5)	1.7 (1.3-2.1)
Northeast	26.3 (26.0-26.5)	7.2 (6.3-8.1)	3.1 (3.0-3.2)	1.3 (1.0-1.6)
East	27.5 (27.3-27.7)	8.7 (8.1-9.3)	3.3 (3.2-3.4)	1.7 (1.5-1.9)
Central	18.4 (18.3-18.5)	5.1 (4.8-5.4)	1.7 (1.6-1.8)	0.8 (0.7-0.9)
South	15.7 (15.6-15.8)	10.0 (9.6-10.4)	1.4 (1.3-1.5)	1.0 (0.9-1.1)
Northwest	16.1 (16.0-16.3)	8.3 (7.9-8.8)	1.5 (1.4-1.6)	1.1 (1.0-1.3)
Southwest	18.4 (18.3-18.6)	4.1 (3.6-4.6)	1.6 (1.5-1.7)	0.4 (0.3-0.6)
**Education**				
Primary school or below	18.3 (18.1-18.6)	4.5 (3.6-5.4)	1.9 (1.8-2.0)	0.5 (0.1-0.9)
Junior high school	21.0 (20.9-21.0)	6.7 (6.4-7.0)	2.2 (2.1-2.3)	1.0 (0.9-1.2)
High school	23.9 (23.7-24.1)	9.6 (8.9-10.2)	2.9 (2.8-3.0)	1.5 (1.2-1.7)
College or higher	30.0 (29.8-30.2)	7.7 (6.9-8.6)	3.6 (3.5-3.7)	1.0 (0.6-1.5)
**GDP level**				
Tertitle 1	21.3 (21.1-21.4)	7.0 (6.6-7.4)	2.4 (2.3-2.4)	1.0 (0.8-1.1)
Tertitle 2	21.9 (21.8-22.0)	7.1 (6.6-7.5)	2.2 (2.1-2.3)	0.9 (0.8-1.1)
Tertitle 3	24.7 (24.6-24.8)	9.1 (8.7-9.5)	3.0 (2.9-3.1)	1.7 (1.5-1.8)

[a]Data are shown as prevalence (95% confidence interval), and all estimates were weighted using the China Population Census in 2010. Overweight and obesity were defined according to the WHO criteria: overweight, 25.0≤ BMI ≤29.9 kg/m^2 and obesity, BMI ≥30.0 kg/m^2.

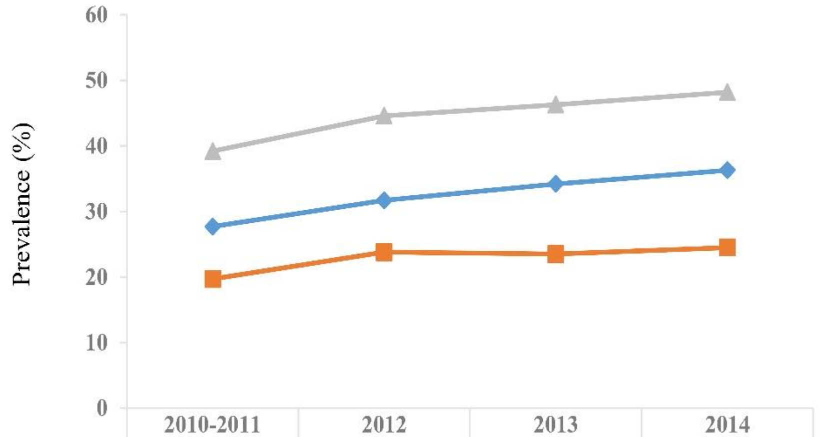

	2010-2011	2012	2013	2014
Men	27.7	31.7	34.2	36.3
Women	19.7	23.8	23.5	24.5
Couples	39.2	44.6	46.3	48.2
